# The Impact of Core Complex Training on Some Basketball-Related Aspects of Physical Strength and Shooting Performance

**DOI:** 10.3390/ejihpe13090118

**Published:** 2023-08-28

**Authors:** Ahmed K. Hassan, Abdulmalek K. Bursais, Mohammed S. Alibrahim, Hossam S. Selim, Ahmed M. Abdelwahab, Badry E. Hammad

**Affiliations:** 1 Department of Physical Education, College of Education, King Faisal University, Al-Ahsa 31982, Saudi Arabia; abursais@kfu.edu.sa (A.K.B.); malibrahim@kfu.edu.sa (M.S.A.); hselim@kfu.edu.sa (H.S.S.); 2 Department of Team Sports and Racket Games, Faculty of Physical Education, Minia University, Minya 61519, Egypt; 3 Seconded to the Department of Fights and Individual Sports, Faculty of Physical Education, Minia University, Minya 61519, Egypt; a.hoba@yahoo.com; 4 Department of Fights and Individual Sports, Faculty of Physical Education, Minia University, Minya 61519, Egypt; badryeidh@mu.edu.eg

**Keywords:** core exercise (CE), complex training (CT), core complex training (CCT), basketball, shooting

## Abstract

Exercises for the core can be categorized as promoting core-stability, core-strength, or functionality, as these are crucial aspects of most sports activities. This study aimed to examine the effects of using core complex training (CCT), complex training (CT), and core exercise (CE) on some aspects of muscle strength and shooting performance in basketball players. The 36 participants were divided into three groups of 12 each, and then the experimental approach was applied to each group. The groups were labeled as follows: the core complex training group (N = 12; age, 18.58 ± 0.67 years; height, 178.08 ± 0.79 cm; weight, 76.42 ± 1.38 kg; training age, 7.42 ± 0.51 years); the complex training group (N = 12; age, 18.50 ± 0.52 years; height, 177.92 ± 1.31 cm; weight, 76.67 ± 1.78 kg; training age, 7.33 ± 0.49 years); and the core exercise group (n = 12; age, 18.42 ± 0.52 years; height, 177.75 ± 1.29 cm; weight, 76.58 ± 1.38 kg; training age, 7.42 ± 0.67 years). For ten weeks, each of the three groups participated in three training sessions every week. This study investigates the impact of core complex training on basketball shooting ability and muscle strength. The eight-week program, consisting of weight training, plyometric exercises, and core exercises, yielded improvements in muscle strength and shooting accuracy. In tests of muscular strength and basketball shooting ability, the CCT group outperformed the CE and CT groups. The F value varied from 3.75 to 58.77, which are function values with a *p* < 0.05 significance level. The core complex training group exhibited superior muscle strength to that of both the complex training group and the core exercise group, in some areas. This is shown in the results of the javelin quadrathlon medicine ball test, the core muscle strength and stability test, the sit-up abdomen test, the sit-up back test, the standing long jump test, the Sargent jump test, and the shooting test (*p* < 0.005). Due to the effect of the core complex training program on improving performance efficiency and muscle strength, which affects the results of matches, we have recommended using the proven basic strength training program at other age stages, with the objective of including the concept, importance, and design of compound basic strength training in training programs used by basketball coaches.

## 1. Introduction

The aim of core complex training is to strengthen the muscles in the core and lower extremities in order to enhance performance and prevent injuries. Core complex training has been demonstrated to improve the knee and lumbar isokinetic muscle function in throwers [1]. The majority of sports actions require a strong core, which can be developed through core-stability, core-strength, and functional workouts [2]. The core musculature is required for both stability and mobility, which allows for the transfer of momentum during athletic movements [3]. Exercises that prioritize isometrics, with imposed loads through limb movement, are frequently included in core complex training programs [4]. These programs can offer physiological and biomechanical benefits that improve preparation for most sports [5].

One of the most important physical traits is muscular strength, since it serves as the basis for movement. The abdominal muscles, gluteus, hip girdle, and pelvic floor muscles make up the core muscles, which work in concert with the muscular trunk to provide the body with strength and stability [6]. The ability to perform motor skills is one of the requirements for success, so some researchers in the field of sports training are interested in discovering new ways, methods, and training strategies to train its components (muscular ability and muscular endurance) to contribute to the growth of physical abilities and increase the level of performance with the least amount of effort and time [7]. The most crucial element of fundamental training is basic endurance since it helps the core muscles maintain the body’s posture effectively [8]. Basic endurance is crucial for spinal stability during extended exercise [9]. Both tall and short basketball players need muscle because it increases speed by a greater percentage than muscle strength does for a tall player. This allows the short player to make up for his lack of height by raising the level of his jump height by increasing the strength of his jump more than the percentage of his speed [10]. In their research findings, Santos and Janeira [11] and Kukuric et al. [12] concluded that mechanically similar plyometric-training should be alternated, group by group, with weight training in the same training session to improve muscular ability and athletic performance. Complex training (CT), which includes weight training and plyometric exercise, is an effective fitness training method, according to Chabut [13], Akuthota [14], Kibler [15], and Tınkır and Uzun [16]. By using strength training (core exercise), which is a reflection of the muscular endurance of the muscles working in the trunk area, a popular form of physical fitness today due to its effectiveness in enhancing performance, the player can raise their performance levels by accelerating their bodies more quickly while in motion.

According to several studies, core exercise (CE) increases the strength and endurance of the trunk muscles, as well as the neuromuscular coordination between the spine and pelvis [17,18,19]. In addition, it helps to facilitate the movement of force from top to bottom and vice versa, which improves the performance of athletic skills [8,15,18,20,21]. Through the studies conducted in the fields of training in general, and basketball in particular, Kukuric et al. [12], Chabut [13], Chaouachi et al. [22], Santos and Janeira [11], Aku-thota et al. [14], and Kibler et al. [15] noted the use of these studies to identify modern methods and strategies for the development of muscle strength components. Basketball is a fast-paced game that calls for swift changes in speed and direction, as well as a fluid rhythm of play that includes quick movements, jumping, and abrupt stops. This makes it necessary to find new and better techniques to enhance physical fitness, particularly the components related to muscle strength [23,24]. In order to provide appropriate recovery from the high-intensity actions involved in the game, recovery tactics are frequently used in basketball. Basketball requires core qualities like agility and reactive agility, not just the ability to complete a straight sprint [25]. Coaches can analyze players’ levels of fitness by comparing them to elite basketball players using standardized, normative data from the NBA Combine Physical Fitness Testing and other tests that are specialized to basketball [26]. We have observed a decline in the proficiency of basketball players in the area of shooting, attributable to certain deficiencies in their physical muscular capacity. In response, we endeavored to employ modern methods to enhance their muscle strength, thereby ameliorating their shooting performance. This study aimed to examine the effects of using core complex training (CCT), complex training (CT), and core exercise (CE) on some aspects of muscle strength and basketball players’ shooting performance. We made the following assumption: (A) there will be significant differences between the (CCT, CE, and CT) group’s pre- and post-measurement averages in several components of muscular strength and the increase in aiming performance., which was confirmed by: (B) the averages of the dimensions measures for the three research groups (CCT, CE, and CT) varied significantly in various aspects of muscular strength and improved aiming performance.

## 2. Materials and Methods

### 2.1. Participation

There were 50 players in the research community, which included basketball players in the Eastern Province of Saudi Arabia. The participants in the study totaled (36) players, who were split into three groups with a total of (12) players in each group. Every group was chosen at random. As can be seen in (Table 1), the three groups were determined to be equal and homogeneous. The following inclusion/exclusion requirements were followed: (1) Basketball players with at least five years’ of experience who participated at the same level. (2) Four athletes were removed because they had injuries that made it difficult for them to perform or shoot baskets. Informed consent forms were supplied to all participants. The King Faisal University’s Scientific Research Ethics Committee in the Kingdom of Saudi Arabia gave the project their stamp of approval. Participants’ health reports were acquired prior to the study’s completion. We use the Steven K. Thompson [27] equation to calculate the sample size, as follows:

$$n=\frac{N\times p(1-p)}{\left[\left[N-1\times \left(d^{2}÷z^{2}\right)\right]+p\left(1-p\right)\right]}$$

n: sample size; N: population size; z: confidence level at 95% (1.96); d: error proportion (0.05); p: proportion (50%).

It is clear from Table 1 that there are no statistically significant differences between the three groups, CCT, CT, CE, which indicates the homogeneity and equivalence of those groups, where *p* > 0.05.

### 2.2. Tools and Devices

We measured weight on a medical scale and height in cm. The InBody 720 tool (InBody Co., Seoul, Republic of Korea) was used to calculate body weight and height. The validity of this instrument has been discussed previously [28,29]. A measuring tape, along with basketballs, a wall stick, an iron bar, stopwatches, various weights, rubber bands, sticks, and Swiss balls were used in the tests. The tests included the core muscle strength and stability (CMSS) test, the sit-up abdomen test (SUA), the sit-up back test, the standing long jump test (SLJ); the Sargent jump test (SJ); the medicine ball javelin quadrathlon (MBJQ) test, and a shooting test (see Appendix B). To ensure the validity and reliability of the measures, the exploratory investigation used a sample different from the original sample.

### 2.3. Procedures

#### 2.3.1. Training Program

The aim of the program is to develop muscle strength and improve the level of shooting performance in basketball. Over a duration of 10 weeks, each of the three groups underwent 30 training units, with each week consisting of 3 training units. The allotted time for each unit ranged from 45 to 60 min, with an additional 15 to 20 min allotted for the warm-up and closure stages, which were conducted separately. The preliminary exercises consisted of general preparation exercises, comprised of 3 to 5 sets, with 8 to 12 repetitions. For the strength stage, the groups were categorized into 3 to 5 groups, with 5 to 6 repetitions. For the stage of strength that was characterized by speed, the groups were divided into 3 to 5 groups, with repetitions ranging from 2 to 5 replications (see Appendix A). Table 2 shows the distribution of the program for the three groups across the days of the week.

#### 2.3.2. Time Frame

Prior to the start of the programs, all research variables were pre-measured for the three groups. Typical warm-ups last for about 15 min. The players were vocally urged to give the tests their highest effort and to concentrate as hard as they could. The research groups’ baseline measurements were taken on 11 and 12 January 2023. Every person in the three groups were tested in the same way, starting on 15 January 2023, and ending on 23 March 2023. On 26 and 27 March 2023, after the training program had been fully implemented, we measured the groups once more in a manner similar to the way in which the pre-measurement was performed.

#### 2.3.3. Statistical Analysis

The results from the 36 participants were taken into account in the statistical analysis using SPSS 26 (IBM, Corp., Armonk, NY, USA). The data exhibited a normal distribution, according to the Kolmogorov–Smirnov test, which we employed to determine their normality, yielding a *p* value of 0.001. To analyze the data, *t*-tests and one-way ANOVA were performed. When d is between 0 and 0.2, the influence is modest or insignificant; when it is between 0.2 and 0.5, the influence is medium; and when it is between 0.5 and 0.8, the influence is strong; beyond 1.4, the influence is quite large. The threshold for significance was set at *p* < 0.05. The significance threshold was set at *p* < 0.05, and the Steven K. Thompson equation was applied to the study population where n: sample size; N: population size; Z: confidence level at 95% (1.96); d: error proportion (0.05); p: percentage (50%), N = (50), *P* = (0.50), d^2^ = (0.0025), and Z^2^ = (3,8416).

## 3. Results

Table 3, Table 4, Table 5 and Table 6 show the results of the before and after measurements for the CCT, CT, and CE groups. The pre- and post-measurements for the CCT group are compared in Table 3, where the calculated F value is greater than the tabular F value at the indicative level. (0.05) (*p* = 0.001). For the tests (CMSS, SUA, SUB, SLJ, SJ, MBJQ, and shooting), the Cohen’s d values were calculated as follows: 3.34, 6.25, 3.46, 7.83, 25.32, 34.80, and 5.61 in the direction of the post-measurement.

The variables for the basketball players in Table 4 are different between the pre- and post-measurements of the CT group because the computed F value is higher than the tabular F value at the indicative level (0.05) (*p* = 0.001). We discovered that the results for the Cohen’s d values for the CMSS, SUA, SUB, SLJ, SJ, MBJQ, and shooting tests revealed the following rates, respectively: 2.60, 4.86, 3.18, 6.73, 11.50, 8.92, and 4.28, in the direction of the post-measurement.

Table 6, where the calculated F value is larger than or equal to the tabular F value at the indicative level, displays the differences between the three groups of basketball players following the measurements. The F value was found to range from 3.75 to 58.77 (*p* < 0.05). The LSD results likewise demonstrated the CCT group’s superiority over the CT and CE groups.

## 4. Discussion

The current study intends to evaluate how some components of basketball shooting ability and muscle strength are affected by core complex training. The training was conducted three times every week for 10 weeks. Due to the effectiveness of CCT, which consists of weight training, plyometric (compound) training, and core exercises, we discovered an improvement in the results of the tests of some components of muscle strength (CMSS, SUA, SUB, SLJ, SJ, and MBJQ) by 14.58%, 42.49%, 31.51%, 12.80%, 30.45%, and 57.63% *p* ≤ 0.05, respectively, and an improvement in the skill of shooting by 76.26. This is because CCT, which includes weight training, plyometric (compound) training, and core exercises, is effective. To develop powerful and evenly distributed muscles all the way around the spine, pelvis, and shoulder girdle bones, this kind of training relies on the integration of body movement as a single unit. The muscles of the lower and upper limbs, as well as the torso, were all significantly strengthened as a result, and there was a noticeable improvement in these muscles. The study by Belli et al. [17] found that an 8-week core training method, using a straightforward exercise program, resulted in discernible improvement. It significantly contributed to the development of the lower leg, trunk, and upper limb muscles. This is consistent with research by Shinkle et al. [30], Byars et al. [31], and Kahle [32] that demonstrated the group of core muscles are responsible for regulating the area surrounding the spine and pelvis, providing stability, balance, and fluid movement for athletes. Movement becomes difficult and the spine becomes unstable without the efficacy of these muscles, which is why completing core workouts is so important. This is in line with the findings of the study by Ingle et al. [33], which showed that incorporating “stretching and shortening” exercises into weight training is beneficial for both the upper and lower body. It is also a secure form of exercise that enhances strength, jumping, throwing, and short-distance running.

According to the research of Santos et al. [11] and Stojanovic. et al. [34], regular weight training for six weeks increased the height and stability of the vertical jump by 3.3 cm, while plyometric training increased it by 3.8 cm. Combining the two types of training over the same time period resulted in an increase of 10.7 cm, confirming the effectiveness of increased muscle mass. Additionally, we discovered improvements in the scores on tests for some muscle strength components (CMSS, SUA, SUB, SLJ, SJ, and MBJQ) *p* ≤ 0.05, as well as an improvement in shooting skill for the (CT) group as a result of the use of complex training We considered the reduction of the time the arms or feet were in contact with the ground while doing the plyometric activities. The “lengthening and shortening” workouts lessen the time it takes for muscles to contract, which is important because the player jumps as soon as his feet or arms hit the ground. This is confirmed by Ebben [35]. In regards to the usefulness of weight training combined with “lengthening and shortening” exercises to increase athletic performance and explosive power, according to research by Ingle et al. [33], combining weight training with “stretching and shortening” exercises is a safe training method that increases strength, leaping, throwing, and short-distance running abilities. In Table 5 of the CE group, we identified improvements in various muscle strength components, *p* ≤ 0.05. This improvement is a result of CE, which was developed to stabilize and strengthen the trunk muscles, which are comprised of 29 muscle pairs [36]. Strengthening this region enhances the motor transport processes between the parties by using fixed muscle contraction exercises to strengthen the internal trunk muscles, create balance and muscular endurance in these muscles, as well as strengthen the abdominal muscle group to increase the strength, rigidity, and bulk of the abdominal muscles. The trunk is the link in the kinetic chain that transfers force from one party to another when performing motor skills, and performing core exercises in various contexts, with varying repetitions and directions, and with varying physical loads, increased both the research sample’s motor stock and motivation for performance. The trunk muscles’ internal and external coordination improved, enabling them to complete the necessary motor tasks, such as running, jumping, passing, and aiming, as well as the quick, sudden movements included in the physical and skill tests being studied.

Strength development benefits from basic exercise [37,38,39,40]. Basic training improves athletic performance, according to numerous studies [19,41,42,43,44]. This is in line with studies by Sharrock et al. [45], Oliver et al. [46], and Saeter-bakken et al. [20] that showed that CE increased players’ physical fitness, which had a significant impact on skill performance and improved aim compared to programs without CE. According to the study by Lee [47], basic stabilization training on an unsteady surface activates the deep abdominal and back muscles and is successful in stabilizing the pelvis and trunk. The CE training course, according to Cissik, [48], Tsukagoshi et al. [49], and Kibler et al. [15], represents a contemporary trend to increase fitness and must be implemented continually to maintain fitness. Because it concentrates on the spine’s strength, stability, and balance—without which it is difficult to transmit movement and performance is subpar—this region is distinctive in its own right. Additionally, it helps to enhance the motor function, speed, and agility of the body, all of which have a significant impact on increasing the level of skill performance owing to a rise in fitness. Maintaining a good baseline of strength and stability might help athletes with their training adaptations, athletic performance, and fitness [8,15,21,50,51,52]. Therefore, it is evident from Table 6 that the CCT group demonstrated superiority over the other groups (CT, CE) in the tests of some muscle strength components (CMSS, SUA, SUB, SLJ, SJ, MBJQ), where the F value showed a significance of 6.33, 25.52, 14.31, 3.75, 31.04, 58.77, *p* < 0.05, respectively, with a correction of 46.19, *p* < 0.05. In the test (30 s abdomen), the results likewise revealed disparities in favor of the CE group and the CT group, and researchers ascribe these variances to the fact that basketball is a sport that requires further development.

Weight training is essential for basketball players, since it enhances their leaping ability, aim, rebounding techniques, and quick passing. Additionally, it extends the shooting range and strengthens the entire body, especially the upper and lower limbs and the torso. Basketball’s defensive and attacking maneuvers necessitate agility to stop, run, change direction, and change speed. Explosive muscle growth is necessary for these motions to be performed effectively, which weight training can help to achieve [53,54,55,56]. The trunk is the link in the kinetic chain that transfers force from one party to another when performing motor skills, and performing core exercises in different contexts, with different repetitions and directions, and with different physical loads, increased the motor stock and motivation for performance in the research sample. The trunk muscles’ internal and external coordination improved, enabling them to carry out the required motor tasks, including running, jumping, passing, and aiming, as well as the quick, sudden movements included in the physical and skill assessments under investigation. The change in training methods, taking into account the individual differences between athletes and appropriate planning for gradation in increasing the load, will also result in reaching the highest levels of performance [21]; thus, the distinguished, innovative coach is the one who designs exercises that have a good impact on improving the capabilities of his players using a variety of sets of exercises within one training unit [57]. And if the coach can rise above the conventional approaches, he will improve the training stimuli and be able to inject significant inspiration, fun, and relief from monotony [24]. The current study’s findings are in line with those of Kumar et al. [58] in that the experimental group that combined training strategies (CT and CE) performed better on the fitness tests than did the groups that used only compound or core training.

### Study Limitations and Strengths

This study demonstrated the impact of core complex training on some basketball-related aspects of physical strength and shooting performance. Basketball players’ physical fitness was evaluated using trustworthy and accurate methodologies. In addition to the fact that the study achieved positive results, it provides supplementary scientific evidence for the usage of compound fundamental workouts. There are certain restrictions, however. When evaluating the data, care must be used because of the tiny sample size, as well as the fact the every participant in the study was male. Additionally, there was no organization in regards to the daily sleeping and eating schedules of the athletes, which would have more completely and precisely characterized the phenomenon and helped the athletes and coaches create more effective training regimens.

## 5. Conclusions

Compound basic strength training exercises have been shown to improve muscle strength and shooting performance in basketball players. The CCT group outperformed the CT and CE groups. Compound fundamental strength exercises should be incorporated into training regimens to increase performance effectiveness and boost match outcomes. It is crucial to remember that based on the age group and individual athlete characteristics, the duration and results of the training program may differ. To improve performance and achieve better outcomes in basketball games, basketball coaches might consider introducing compound fundamental strength training exercises into their programs.

## Figures and Tables

**Table 1:** Mean, deviation, and F value for age, height, weight, and training age.

Variables	Standard	CCT N = 12		CT N = 12		CE N = 12		F	Sig.
		Mean	SD	Mean	SD	Mean	SD		
Age	Year	18.58	0.67	18.50	0.52	18.42	0.51	0.254	0.777
Height	cm	178.08	0.79	177.92	1.31	177.75	1.29	0.250	0.781
Weight	kg	76.42	1.38	76.67	1.78	76.58	1.38	0.084	0.920
Training age	Year	7.42	0.51	7.33	0.49	7.42	0.67	0.087	0.917

**Table 2:** The time table for implementing the three training programs.

Groups	Program	Saturday	Sunday	Monday	Tuesday	Wednesday	Thursday
First	CCT		** 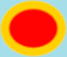 **		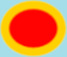		** 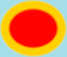 **
Second	CT	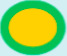		** 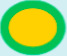 **		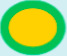	
Third	CE		** 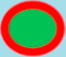 **		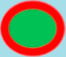		** 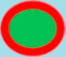 **

**Table 3:** The mean and standard deviation pre- and post-test for the basketball players in the CCT group.

Variables	Standard	PRE		POST		95% Confidence Interval of the Difference		t	Cohen’s d	Imp. Percent	Sig
		Mean	Std. Deviation	Mean	Std. Deviation	Lower	Upper				
CMSS	min	1.92	0.08	2.20	0.05	−0.33	−0.22	11.57	3.34	14.58	0.001 *
SUA	count	18.83	0.72	26.83	0.94	−8.81	−7.19	21.66	6.25	42.49	0.001 *
SUB	count	17.17	0.72	22.58	1.51	−6.41	−4.42	12.00	3.46	31.51	0.001 *
SLJ	cm	196.58	3.58	221.75	2.26	−27.21	−23.12	27.12	7.83	12.80	0.001 *
SJ	cm	37.04	0.19	48.32	0.36	−11.56	−11.00	87.72	25.32	30.45	0.001 *
MBJQ	meter	5.24	0.07	8.26	0.06	−3.08	−2.97	120.55	34.80	57.63	0.001 *
Shooting	count	7.33	0.49	12.92	1.00	−6.22	−4.95	19.42	5.61	76.26	0.001 *

CMSS = core muscle strength and stability; SUA = sit-up abdomen test; SUB = sit-up back test; SLJ = standing long jump test; SJ = Sargent jump test; MBJQ = medicine ball javelin quadrathlon test; SD = std. deviation; Imp. percent = improvement percent; * *p* < 0.05.

**Table 4:** The mean and standard deviation pre- and post-test for the basketball players in the CT group.

Variables	Standard	PRE		POST		95% Confidence Interval of the Difference		t	Cohen’s d	Imp. Percent	Sig
		Mean	SD	Mean	SD	Lower	Upper				
CMSS	min	1.91	0.06	2.15	0.06	−0.30	−0.18	9.02	2.60	12.57	0.001 *
SUA	count	18.33	0.78	24.67	0.89	−7.16	−5.51	16.84	4.86	34.59	0.001 *
SUB	count	17.08	0.52	20.75	0.75	−4.40	−2.93	11.00	3.18	21.49	0.001 *
SLJ	cm	194.83	3.54	220.00	1.71	−27.54	−22.79	23.33	6.73	12.92	0.001 *
SJ	cm	37.01	0.14	46.38	0.80	−9.89	−8.85	39.83	11.50	25.32	0.001 *
MBJQ	meter	5.19	0.06	7.33	0.25	−2.29	−1.98	30.90	8.92	41.23	0.001 *
Shooting	count	7.25	0.62	10.58	0.52	−3.83	−2.84	14.83	4.28	45.93	0.001 *

CMSS = core muscle strength and stability; SUA = sit-up abdomen test; SUB = sit-up back test; SLJ = standing long jump test; SJ = Sargent jump test; MBJQ = medicine ball javelin quadrathlon test test; SD = std. deviation; Imp. percent = improvement percent; * *p* < 0.05.

**Table 5:** The mean and standard deviation pre- and post-test for the basketball players in the CE group.

Variables	Standard	PRE		POST		95% Confidence Interval of the Difference		t	Cohen’s d	Imp. Percent	Sig
		Mean	SD	Mean	SD	Lower	Upper				
CMSS	min	1.90	0.05	2.12	0.05	−0.26	−0.17	10.81	3.12	11.58	0.001 *
SUA	count	18.42	0.52	24.58	0.79	−6.93	−5.41	17.90	5.17	33.44	0.001 *
SUB	count	17.17	0.72	20.58	0.52	−4.05	−2.78	11.88	3.43	19.86	0.001 *
SLJ	cm	194.75	3.55	219.92	1.51	−27.46	−22.87	24.12	6.96	12.92	0.001 *
SJ	cm	37.01	0.13	46.26	0.89	−9.79	−8.71	37.75	10.90	24.99	0.001 *
MBJQ	meter	5.21	0.05	7.38	0.32	−2.37	−1.97	24.11	6.96	41.65	0.001 *
Shooting	count	7.17	0.72	10.42	0.52	−3.73	−2.77	14.94	4.31	45.33	0.001 *

CMSS = core muscle strength and stability; SUA = sit-up abdomen test; SUB = sit-up back test; SLJ = standing long jump test; SJ = Sargent jump test; MBJQ = medicine ball javelin quadrathlon test; SD = std. deviation; Imp. percent = improvement percent; * *p* < 0.05.

**Table 6:** The mean, SD, and F-values for the three groups of basketball players (CCT, CT, and CE).

Dependent Variable	Standard	Groups		LSD			Mean	SD	F	Sig.
				Mean Difference	Std. Error	Sig.				
CMSS	min	CCT	CT	0.048 *	0.0218	0.036	2.20	0.05	6.33	0.005 *
			CE	0.077 *	0.0218	0.001				
		CT	CCT	0.048*	0.0218	0.036	2.15	0.05		
			CE	0.029	0.0218	0.189				
		CE	CCT	0.077 *	0.0218	0.001	2.12	0.05		
			CT	0.029	0.0218	0.189				
SUA	count	CCT	CT	2.167 *	0.357	0.001	26.83	0.94	25.52	0.001 *
			CE	2.250 *	0.357	0.001				
		CT	CCT	2.167 *	0.357	0.001	24.67	0.89		
			CE	0.083	0.357	0.817				
		CE	CCT	2.250 *	0.357	0.001	24.58	0.79		
			CT	0.083	0.357	0.817				
SUB	count	CCT	CT	1.833 *	0.415	0.001	22.58	1.51	14.31	0.001 *
			CE	2.000 *	0.415	0.001				
		CT	CCT	1.833 *	0.415	0.001	20.75	0.75		
			CE	0.167	0.415	0.690				
		CE	CCT	2.000 *	0.415	0.001	20.58	0.51		
			CT	0.167	0.415	0.690				
SLJ	cm	CCT	CT	1.750 *	0.756	0.027	221.75	2.26	3.75	0.034 *
			CE	1.833 *	0.756	0.021				
		CT	CCT	1.750 *	0.756	0.027	220.00	1.71		
			CE	0.083	0.756	0.913				
		CE	CCT	1.833 *	0.756	0.021	219.92	1.51		
			CT	0.083	0.756	0.913				
SJ	cm	CCT	CT	1.943 *	0.294	0.001	48.32	0.36	31.04	0.001 *
			CE	2.064 *	0.294	0.001				
		CT	CCT	1.943 *	0.294	0.001	46.38	0.80		
			CE	0.121	0.294	0.684				
		CE	CCT	2.064 *	0.294	0.001	46.26	0.89		
			CT	0.121	0.294	0.684				
MBJQ	meter	CCT	CT	0.928 *	0.096	0.001	8.26	0.06	58.77	0.001 *
			CE	0.877 *	0.096	0.001				
		CT	CCT	0.928 *	0.096	0.001	7.33	0.25		
			CE	0.051	0.096	0.601				
		CE	CCT	0.878 *	0.096	0.000	7.38	0.32		
			CT	0.051	0.096	0.601				
Shooting	count	CCT	CT	2.333 *	0.291	0.001	12.92	1.00	46.19	0.001 *
			CE	2.500 *	0.291	0.001				
		CT	CCT	2.333 *	0.291	0.001	10.58	0.51		
			CE	0.167	0.291	0.571				
		CE	CCT	2.500 *	0.291	0.001	10.42	0.51		
			CT	0.167	0.291	0.571				

CMSS = core muscle strength and stability; SUA = sit-up abdomen test; SUB = sit-up back test; SLJ = standing long jump test; SJ = Sargent jump test; MBJQ = medicine ball javelin quadrathlon test; SD = std. deviation; Imp. percent = improvement percent; * *p* < 0.05.

**Figure A1:**
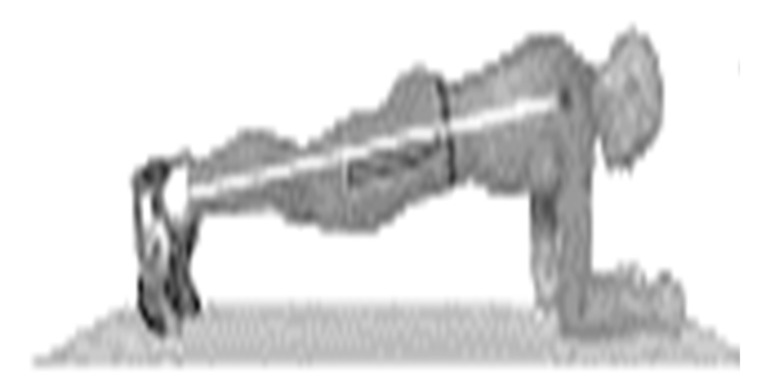
Core muscle strength and stability test.

**Figure A2:**
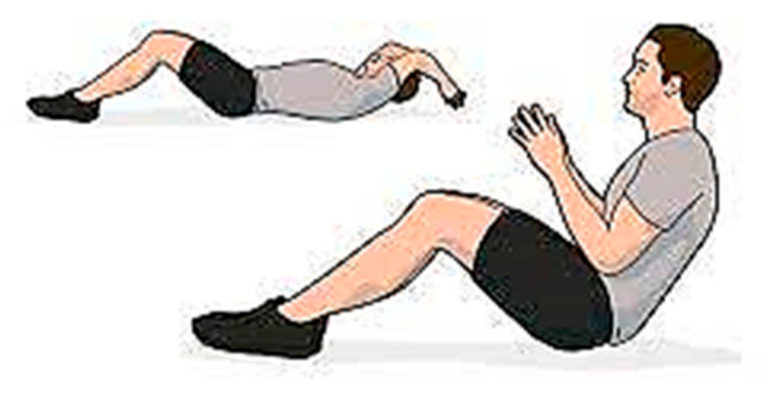
Sit-up abdominal test.

**Figure A3:**
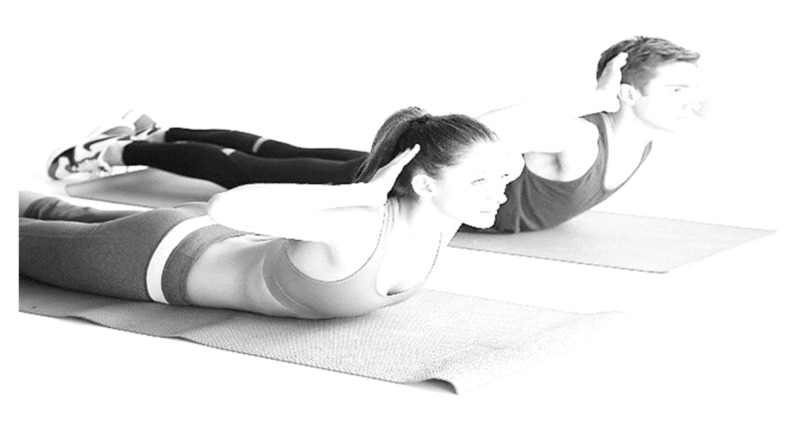
Sit-up back test.

**Figure A4:**
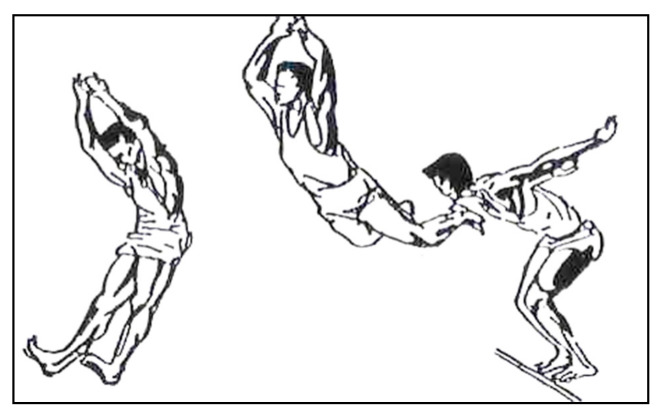
Standing long jump test.

**Figure A5:**
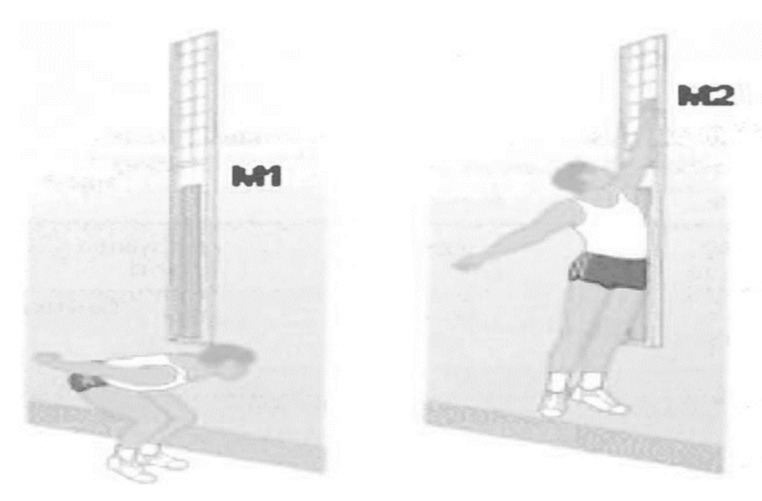
Sargent jump test.

**Figure A6:**
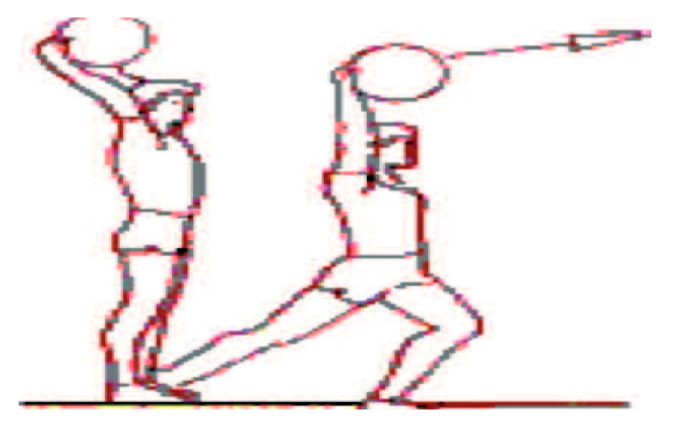
Standing throw.

**Figure A7:**
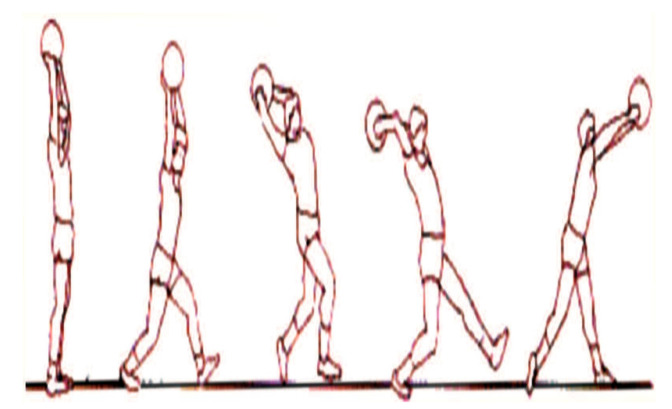
Three-step throw.

**Figure A8:**
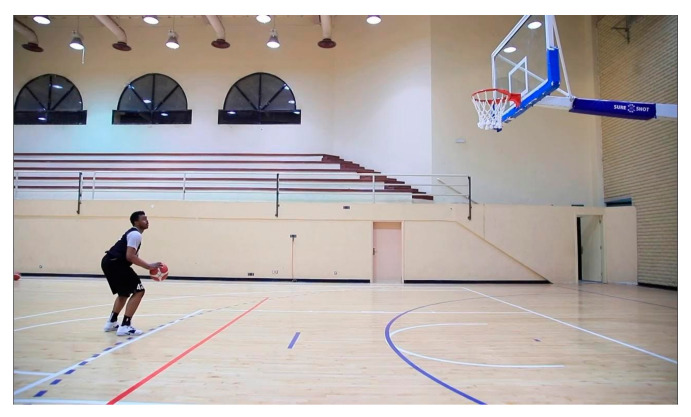
Shooting performance test.

## Appendix A

Examples of some of the workouts included in the program. The appendix contains descriptions of the three training programs (core complex training; core exercise training; complex training).

**Table A1:** Core complex training (CCT).

No.	Exercise	Number of Groups	Repetition per Group	Intergroup Rest	Inter-Rest
1	- Standing, carrying the bar in front of the body, with the palm of the hand outward, bending the arms up. - Standing, carrying a medicine ball, throw the medicine ball back.	3 3	9 10	2 s 2 s	4–5 min
2	While prostrate, clasp the hands behind the head, raising and lowering the chest.	3	30 s	30 s	1–2 min
3	- Squatting. Extend my knees - Deep jump.	3 3	11 8	2 s 2 s	4–5 min
4	Prostrate, forearms on the ground, moving forward and backward, using the wheel.	3	30 s	30 s	1–2 min
5	- Standing, carry the weight on the shoulders behind the neck bend the knees in half.- Bend the knees in half and complete the vertical jump upwards.	3 3	10 10	2 s 2 s	4–5 min
6	Inverse oblique prone in front at a man’s height; maintain stability (repeat for other leg).	3	30 s	30 s	1–2 min
7	- Standing open, flex, carrying the weight on the shoulders, jump high.- Glass jump over the cones with both feet.	3 3	10 8 round-trip and 4 return	2 s 2 s	4–5 min
8	Lateral prostration, lower leg bent 60 degrees. Lower arm high and the other bent in front of the chest, palm on the floor. Lower the subordinate muscles, with one leg swinging forward and backward. (repeat with other leg).	3	30 s	30 s	1–2 min
9	- Standing, with bar held behind the neck and elbow up, stretch the forearm up.- Standing still, hold a medicine ball behind the neck and throw the medicine ball forward and up.	3 3	10 12	2 s 2 s	4–5 min

**Table A2:** Core exercises (CE).

No.	Exercises
1	Transverse’s Abdominals (TA) Activation
2	Fall-Out Exercises
3	Bridging Exercises
4	Planking Exercises
5	Abdominal Exercises
6	Extension Exercise
7	Lunging Exercises
8	Leaning Exercises
9	Cat Curl Exercises
10	Medicine Ball Exercises
11	Pull-Up Exercises
12	Twisting Exercises
13	Push-Up Exercises
14	Opposite Arm and Leg Exercises
15	Stretching Exercises

**Table A3:** Exercises for the abdominal muscles that correlate with the warm-up exercises.

Method of performance	- From a backwards-lying position with the fingers 2 cm below and to the side of the hip joint, pull the abdomen down from the belly button with a force of 30%, and retain muscle contraction for (10) seconds.	
Iteration	10 iterations	
Groups	1–3 sets	
Rest	30–90 s	
- 2 -		
Method of performance	- Lying on the back pulling the abdomen down from the navel area with a force of 30%, maintain a muscle contraction while raising one leg, remain motionless, then return to your starting position and repeat with the other leg. - To improve performance, you can lift one leg high and hold it there while raising the other leg at the same position and bringing each leg back to the ground individually.	
Iteration	20 repetitions	
Groups	1–3 sets	
Rest	30–90 s	
- 3 -		
Method of performance	- Lie on the abdomen, without movement, and pull the abdomen towards the pelvic floor from the navel area with a contraction force of 30%.	
Iteration	10 iterations	
Groups	1–3 sets	
Rest	30–90 s	
- 4-		
Method of performance	- Hands below the shoulders and knees below the hips. Pull the abdomen towards the pelvic floor from the belly button area with a force of 30%.	
Iteration	10 iterations	
Groups	1–3 sets	
Rest	30–90 s	
- 5-		
Method of performance	- Hands below the shoulders and knees below the hips. - Pull the abdomen towards the pelvic floor from the belly button area with a force of 30%. Lift the knee off the ground and hold steady (10–15) seconds and then return to the ground.	
Iteration	10 iterations	
Groups	1–3 sets	
Rest	30–90 s	

**Table A4:** Samples of fall-out exercises for the core training program.

- 1 -		
Method of performance	- Lie on the back with the knees and feet together. - Pull the abdomen towards the pelvic floor from the belly button. - Slowly move the knees to the side 3 cm, while keeping the body firm. - Return to the middle, and then repeat on the other side of the body.	
Iteration	10 repetitions per side.	
Groups	1–3 sets	
Rest	30–90 s	
- 2 -		
Method of performance	- Lie on your back with your feet up and together. - Pull the abdomen towards the pelvic floor from the belly button. - Slowly move the knees to the side 3 cm, while keeping the body firm. - Return to the middle, and then repeat on the other side of the body.	
Iteration	10 repetitions per man	
Groups	1–3 sets	
Rest	30–90 s	
- 3 -		
Method of performance	- Lie on the back with feet up and a medicine ball placed between the knees. - Pull the abdomen towards the pelvic floor from the belly button. - Slowly move the knees to the side 3 cm, while keeping the body firm. - Return to the middle, and then repeat on the other side of the body.	
Iteration	10 iterations	
Groups	1–3 sets	
Rest	30–90 s	
- 4 -		
Method of performance	While lying on the back, as shown in the picture, allow one of the legs to fall aside, while maintaining the stability of the other side on the ground in the presence of the weight.	
Iteration	15 repetitions	
Groups	5 sets per leg	
Rest	30–90 s	
- 5 -		
Method of performance	While lying on the back, as shown in the picture, allow the two legs to fall together to the right side, while maintaining the stability of the other side on the ground in the presence of the weight.	
Iteration	15 repetitions per side	
Groups	Two sets per side	
Rest	30–90 s	

**Table A5:** Complex training (CT).

No.	Exercise Description	Groups	Group Replication	Rest between Groups	Rest between Weight Training and Barometric
1	Standing, hold the bar in front of the body, with the palm of the hand outward, bending the arms up. Standing, carrying a medicine ball, throw the medicine ball back.	3 3	9 10	2 s 2 s	4–5 min
2	Squatting, stretching the knees, deep jump from above a box.	3 3	11 8	2 s 2 s	4–5 min
3	Standing, hold the bar behind the neck with elbows up and extend the forearms up. Standing still, hold a medicine ball behind the neck, and throw the medicine ball back and up.	3 3	9 10	2 s 2 s	4–5 min
4	Standing, carry the weight on the shoulders behind the neck and bend the knees in half. Bend the knees half while completing the vertical jump.	3 3	10 10	2 s 2 s	4–5 min
5	Lying down, with the arms high and parallel to the floor, hold the bar in front of the chest. Standing in a comfortable position, holding 5 kg dumbbells in each hand, swing one arm forward and up to head level and the other arm backward.	3 3	10 12	2 s 2 s	4–5 min
6	(Standing, flex, carrying the weight on the shoulders, and jump high. Zigzag jump over the cones with both feet.	3 3	10 8 round-trip and 4 return	2 s 2 s	4–5 min
7	Standing, carrying the weight in front of the body, bend the torso in front of the bottom, stretching and bending the arms, pass a 3 kg medicine ball from the chest to a colleague	3 3	10 10	2 s 2 s	4–5 min

**Table A6:** Core Complex training (CCT).

NO.	Exercise	Number of Groups	Repetition per Group	Intergroup Rest	Inter-Rest	Reviews
1	- Standing, casrrying the bar in front of the body with the palm of the hand outward, bend the arms up. - Standing, carrying a medical ball, throw the medicine ball back.	3 3	9 10	2 s 2 s	4 to 5 min	
2	While prostrate, clasp the hands behind the head, raising and lowering the chest.	3	30 s	30 s	1 s to 2 s	
3	- Squatting, stretch the knees and complete a deep jump.	3 3	10 8	2 s 2 s	4 to 5 min	
4	While prostrate, with the forearms on the ground, move forward and backward, using the wheel.	3	30 s	30 s	1 s to 2 s	
5	- Standing, carry the weight on the shoulders behind the neck and bend the knees in half. - Bend the knees in half and complete the vertical jump upwards.	3 3	10 10	2 s 2 s	4 to 5 min	
6	Inverse oblique, prone in front at the man’s height). Maintain stability. (Repeats for other leg.)	3	30 s	30 s	1 s to 2 s	
7	- Standing, flex while carrying weight on the shoulders, and jump high. - Glass jump over the cones with both feet.	3 3	10 8 round-trip and 4 return	2 s 2 s	4 to 5 min	
8	While laterally prostrate, with the lower leg bent at 60 degrees, with the lower the arm high and the other bent in front of the chest, with the palm on the floor, lower the subordinate muscles, with one leg swinging forward and backward. (Repeats forother leg.)	3	30 s	30 s	1 s to 2 s	
9	- Standing, hold the bar behind the neck with elbows up, stretching the forearm up. - While standing still, place a medicine ball behind the neck and throw the medicine ball forward and up.	3 3	10 12	2 s 2 s	4 to 5 min	

## Appendix B

This appendix contains a description of the physical and skill tests for the basketball players

**Core Muscle Strength and Stability Test:**

The Core Muscle Strength Test is a tool used to track an athlete’s core strength growth. It involves holding the back, neck, and head in a stance, with the helper starting the watch after the correct position is believed to be achieved. The test involves several stages, including the Chinese Push-Up stance, which involves holding the correct position for 1 min. The test is discontinued if the athlete cannot maintain the correct position. The test is completed by tracking the point at which the athlete is unable to maintain the proper body position or cannot proceed for 3 min [59].

The growth of the athlete’s core strength can be tracked with the Core Muscle Strength Test. You will need a flat surface, a helper, a mat or other object to support the elbows and arms, as well as a watch, to complete this test. The back, neck, and head should be held in the stance shown in Figure A5 while the test is being administered. The test is to be discontinued if the athlete is unable to maintain this position. The following describes the method by which the core muscle strength test is carried out: Stage 1. Assume the Chinese Push-Up stance, as shown in Figure A1, by using the mat to support your elbows and arms. The helper starts the stopwatch after the correct position is believed to be achieved. Hold this posture for 1 min. Stage 2: Extend your right arm upward. For 15 s, maintain this posture. Stage 3: Lower your right arm to the floor while raising your left arm. For 15 s, maintain this posture. Stage 4: Lower your left arm to the floor while raising your right leg. For 15 s, maintain this posture. Stage 5: Lower your left leg to the floor while raising your right leg. For 15 s, maintain this posture. Stage 6: Extend your right arm and left leg above the ground. For 15 s, maintain this posture. Step 7: Lower your right arm and left leg to the ground. Raise your left arm and right leg off the ground. For 15 s, maintain this posture. Stage 8. Get back into the fundamental Chinese Push-Up stance. For 30 s, maintain this posture. Stage 9: Test completion. Keep track of the point during the test at which the athlete is unable to maintain the proper body position or cannot proceed.

**Sit-Up Test:**
**Abdominal Test:**

This test monitors the athlete’s abdominal muscle development using a sit-up test. Participants lie on a mat, bend their knees, and raise their feet. A buddy supports them, and the number of sit-ups completed in 30 s is recorded [59].

The objective of this test is to monitor the development of the athlete’s abdominal muscles. You will need a flat surface, a mat, and a partner to hold the feet in order to complete this exam. The sit-up test is conducted as follows: Begin each sit-up with your back on the mat, knees bent, feet flat on the ground, and arms folded across your chest. Return to the floor after raising yourself to a 90-degree angle. A buddy can support the feet. Record the number of sit-ups completed in 30 s.

**Back test:**

This test measures an athlete’s back muscle growth by raising their torso and returning to the starting position with their feet fixed. It involves lying prone on a mat, using a partner for foot support, and counting the number of torso rises completed in 30 s [60].

The objective of this test is to keep track of how the athlete’s back muscles are growing. To do this test, you will need a mat, a flat surface, and a partner to hold the feet still. The test is conducted as follows: the athlete lies fully prone on his stomach with his hands clasped behind his head. When the start signal is given, the athlete raises his torso and returns to the starting position with the feet fixed. The test is complete when the second signal is given. The companion can help provide foot support. Count how many torso rises you were able to complete in 30 s.

**Standing Long Jump Test**

This test measures an athlete’s improvement in elastic leg strength using a 30 m tape measure, a long jump pit, and an assistant. The athlete jumps into the pit while crouching, bending forward, swinging the arms backward, and leaning forward [60].

The objective of this test is to track the athlete’s improvement in elastic leg strength. To complete this test, you will need: a 30 m tape measure, a long jump pit, and an assistant. The athlete extends his feet beyond the sandpit’s edge. The athlete jumps into the sandpit with both feet while crouching, bending forward, swinging the arms backward, and leaning forward. The coach should take a measurement from the sandpit’s edge to the point of contact that is closest to the take-off point. The jump must begin from a stationary posture.

**Sargent Jump Test**

This test aims to track an athlete’s improvement in elastic leg strength using a wall, a 1 m tape measure, chalk, and an assistant. The athlete applies chalk to the fingernails, stands sideways, reaches up, and marks the wall (M1). The coach calculates the separation between M1 and M2, and the athlete can take the test multiple times [59].

The objective of this test is to track the athlete’s improvement in elastic leg strength. To complete this test, you will need: a wall, a 1 m tape measure, chalk, and an assistant. The athlete applies chalk to the tips of his fingernails, stands sideways against the wall with both feet firmly planted, reaches up as high as possible with one hand, and uses the tips of his fingers to mark the wall (M1); from a standing position, the athlete then jumps as high as possible; and marks the wall with his chalked fingernails. (M2). The coach calculates the separation between M1 and M2. The athlete is free to take the test as many times as desired.

**Medicine Ball Javelin Quadrathlon:**

This test measures an athlete’s strength and fitness progress using a 30 m tape measure; 1.5 kg, 2 kg, and 3 kg medicine balls; and an assistant. It involves four throws: standing, standing, stepping, and stepping. The athlete makes each throw, and the helper records the distance covered. The distance is measured from the front foot to the ball’s landing spot [59].

The objective of this test is to keep tabs on how the athlete’s strength and fitness are progressing. To do this test, you will need a 30 m tape measure; 1.5 kg, 2 kg, and 3 kg medicine balls; and an assistant. The test consists of three medicine ball throws: the first standing throw (men 2 kg, women 1.5 kg), the second standing throw (men 3 kg, women 2 kg), and the third step throw (men 2 kg, women 1.5 kg). Each throw is made by the athlete, and the helper logs the distance covered.

The following throws are explained: standing throw—face forward while holding the medicine ball in both hands, above your head. The feet should be parallel, toeing the line of measurement. Throw the ball for distance, A follow through step is allowed. Distance is measured from the front foot (on release) to where the ball lands. Three-step throw—start in a stationary position with both feet together for a. Taking two steps forward while holding the medicine ball in each hand; if you want to throw the ball far, a final step is permissible. The distance is measured from the front foot’s release point to the spot where the ball lands.

**Shooting Performance:**

After the warm-up, the player takes a shot at the basket while standing behind the free-throw line. With a three-minute pause in between each set, each player takes three sets of five free throws. The players under the basket passes the ball to the referee. After the first player has taken five shots at the basket, the second player follows suit. For the test calculation, the number of correct throws was used for all three groups [61,62].

## References

[1] Lee M., Han G. (2016). The effect of peculiar complex core balance training on isokinetic muscle functions of the knee and lumbus. J. Phys. Ther. Sci..

[2] Jeong Y., Hwang M., Sung W. (2020). Dataset Distillation for Core Training Set Construction. Stat. Methods Appl..

[3] Chang K.-W., Srikumar V., Roth D., Blockeel H., Kersting K., Nijssen S., Železný F. (2013). Multi-core Structural SVM Training. Machine Learning and Knowledge Discovery in Databases.

[4] Judge L.W. (2008). Core Training for Superior Sports Preparation. J. Coach. Educ..

[5] Mattacola C.G., Kiesel K., Burton L., Cook G. (2004). Mobility Screening for the Core. Athl. Ther. Today.

[6] Willson J.D., Dougherty C.P., Ireland M.L., Davis I.M. (2005). Core Stability and Its Relationship to Lower Extremity Function and Injury. J. Am. Acad. Orthop. Surg..

[7] Anant S.K., Venugopal R. (2020). Effect of eight-week core muscles strength training on physical fitness and body composition variables in male players of team games. Rev. Andal. Med. Deport..

[8] Sanchez-Munoz C., Sanz D., Zabala M. (2007). Anthropometric characteristics, body composition and somatotype of elite junior tennis players. Br. J. Sport. Med..

[9] Barati A., SafarCherati A., Aghayari A., Azizi F., Abbasi H. (2013). Evaluation of Relationship between Trunk Muscle Endurance and Static Balance in Male Students. Asian J. Sport. Med..

[10] Stølen T., Chamari K., Castagna C., Wisløff U. (2005). Physiology of Soccer. Sport. Med..

[11] Santos E.J.A.M., Janeira M.A.A.S. (2012). The Effects of Resistance Training on Explosive Strength Indicators in Adolescent Basketball Players. J. Strength Cond. Res..

[12] Kukuric A., Karalejic M., Petrovic B., Jakovljevic S. (2009). Effect of Complex Training on Explosive Strength of Legs Extensors in Junior Basketball Players. Phys. Cult..

[13] Chabut L. (2009). Core Strength for Dummies.

[14] Akuthota V., Ferreiro A., Moore T., Fredericson M. (2008). Core Stability Exercise Principles. Curr. Sport. Med. Rep..

[15] Ben Kibler W., Press J., Sciascia A. (2006). The Role of Core Stability in Athletic Function. Sport. Med..

[16] Tınkır D.A., Uzun A. (2022). The Effect of Vertical and Horizontal Core Trainings on Core Strength, Agility and Speed. Turk. J. Sport Exerc..

[17] Belli G., Marini S., Mauro M., Latessa P.M., Toselli S. (2022). Effects of Eight-Week Circuit Training with Core Exercises on Performance in Adult Male Soccer Players. Eur. J. Investig. Health Psychol. Educ..

[18] Willardson J.M. (2008). A periodized approach for core training. ACSM’s Health Fit. J..

[19] Prieske O., Muehlbauer T., Borde R., Gube M., Bruhn S., Behm D.G., Granacher U. (2016). Neuromuscular and athletic performance following core strength training in elite youth soccer: Role of instability. Scand. J. Med. Sci. Sport..

[20] Saeterbakken A.H., Van Den Tillaar R., Seiler S. (2011). Effect of Core Stability Training on Throwing Velocity in Female Handball Players. J. Strength Cond. Res..

[21] Hibbs A.E., Thompson K.G., French D., Wrigley A., Spears I. (2008). Optimizing Performance by Improving Core Stability and Core Strength. Sport. Med..

[22] Chaouachi A., Brughelli M., Chamari K., Levin G.T., Ben Abdelkrim N., Laurencelle L., Castagna C. (2009). Lower Limb Maximal Dynamic Strength and Agility Determinants in Elite Basketball Players. J. Strength Cond. Res..

[23] Gál-Pottyondy A., Petró B., Czétényi A., Négyesi J., Nagatomi R., Kiss R.M. (2021). Collection and Advice on Basketball Field Tests—A Literature Review. Appl. Sci..

[24] Hassan A.K., Alhumaid M.M., Hamad B.E. (2022). The Effect of Using Reactive Agility Exercises with the FITLIGHT Training System on the Speed of Visual Reaction Time and Dribbling Skill of Basketball Players. Sports.

[25] Huyghe T., Calleja-Gonzalez J., Terrados N. (2020). Post-Exercise Recovery Strategies in Basketball: Practical Applications Based on Scientific Evidence. Basketball Sports Medicine and Science.

[26] González J.M.M., Guerra Y.d.S., García-Manso J.M., Arriaza E. (2016). Design and flow in basketball. Int. J. Heat Technol..

[27] Thompson S.K. (2012). Estimating Proportions, Ratios, and Subpopulation Means.

[28] Stanković M., Čaprić I., Đorđević D., Đorđević S., Preljević A., Koničanin A., Maljanović D., Nailović H., Muković I., Jelaska I. (2023). Relationship between Body Composition and Specific Motor Abilities According to Position in Elite Female Soccer Players. Int. J. Environ. Res. Public Health.

[29] Gonzalez M.C., Orlandi S.P., Santos L.P., Barros A.J. (2018). Body composition using bioelectrical impedance: Development and validation of a predictive equation for fat-free mass in a middle-income country. Clin. Nutr..

[30] Shinkle J., Nesser T.W., Demchak T.J., McMannus D.M. (2012). Effect of Core Strength on the Measure of Power in the Extremities. J. Strength Cond. Res..

[31] Byars A., Gandy-Moodie N., Greenwood L., Stanford M.S., Greenwood M. (2011). An Evaluation of the Relationships Between Core Stability, Core Strength, and Running Economy in Trained Runners. J. Strength Cond. Res..

[32] Kahle N. (2009). The Effects of Core Stability Training on Balance Testing in Young, Healthy Adults. Undergraduate Thesis.

[33] Ingle L., Sleap M., Tolfrey K. (2006). The effect of a complex training and detraining programme on selected strength and power variables in early pubertal boys. J. Sport. Sci..

[34] Stojanovic M.D., Ostojic S., Gonzalez J.M.C., Milosevic Z., Mikic M. (2012). Correlation between explosive strength, aerobic power and repeated sprint ability in elite basketball players. J. Sport. Med. Phys. Fit..

[35] Ebben W.P. (2002). Complex training: A brief review. J Sport. Sci. Med..

[36] Jeffreys I. (2002). Developing a Progressive Core Stability Program. Strength Cond. J..

[37] Alizamani S., Ghasemi G., Nejadian S.L. (2023). Effects of eight week core stability training on stable- and unstable-surface on ankle muscular strength, proprioception, and dorsiflexion in athletes with chronic ankle instability. J. Bodyw. Mov. Ther..

[38] Leetun D.T., Ireland M.L., Willson J.D., Ballantyne B.T., Davis I.M. (2004). Core Stability Measures as Risk Factors for Lower Extremity Injury in Athletes. Med. Sci. Sport. Exerc..

[39] McGill S.M. (2001). Low Back Stability: From Formal Description to Issues for Performance and Rehabilitation. Exerc. Sport Sci. Rev..

[40] Durall C.J., Udermann B.E., Johansen D.R., Gibson B., Reineke D.M., Reuteman P. (2009). The Effects of Preseason Trunk Muscle Training on Low-Back Pain Occurrence in Women Collegiate Gymnasts. J. Strength Cond. Res..

[41] Sato K., Mokha M. (2009). Does Core Strength Training Influence Running Kinetics, Lower-Extremity Stability, and 5000-m Performance in Runners?. J. Strength Cond. Res..

[42] Afyon Y.A., Mulazimoglu O., Boyacı A. (2017). The Effects of Core Trainings on Speed and Agility Skills of Soccer Players. Int. J. Sport. Sci..

[43] Sever O., Zorba E. (2018). Comparison of effect of static and dynamic core exercises on speed and agility performance in soccer players. Isokinet. Exerc. Sci..

[44] Taskin C. (2016). Effect of Core Training Program on Physical Functional Performance in Female Soccer Players. Int. Educ. Stud..

[45] Sharrock C., Cropper J., Mostad J., Johnson M., Malone T. (2011). A pilot study of core stability and athletic performance: Is there a relationship?. Int. J. Sport. Phys. Ther..

[46] Oliver G.D., Dwelly P.M., Sarantis N.D., Helmer R.A., Bonacci J.A. (2010). Muscle Activation of Different Core Exercises. J. Strength Cond. Res..

[47] Lee K. (2023). Motion Analysis of Core Stabilization Exercise in Women: Kinematics and Electromyographic Analysis. Sports.

[48] Cissik J.M. (2011). The Role of Core Training in Athletic Performance, Injury Prevention, and Injury Treatment. Strength Cond. J..

[49] Tsukagoshi T., Shima Y., Nakase J., Goshima K., Takahashi R., Aiba T., Yoneda Y., Moriyama S., Kitaoka K. (2011). Relationship between core strength and balance ability in high school female handball and basketball players. Br. J. Sport. Med..

[50] Borghuis J., Hof A.L., Lemmink K.A.P.M. (2008). The Importance of Sensory-Motor Control in Providing Core Stability: Implications for Measurement and Training. Sport. Med..

[51] Luo S., Soh K.G., Soh K.L., Sun H., Nasiruddin N.J.M., Du C., Zhai X. (2022). Effect of Core Training on Skill Performance Among Athletes: A Systematic Review. Front. Physiol..

[52] Zemková E., Zapletalová L. (2022). The Role of Neuromuscular Control of Postural and Core Stability in Functional Movement and Athlete Performance. Front. Physiol..

[53] Türker A., Yüksel O. (2020). The effect of functional and supportive classic strength trainings in basketball players on aerobic strength, dynamic balance and body composition. Pedagog. Phys. Cult. Sport..

[54] Hermassi S., Schwesig R., Aloui G., Shephard R.J., Chelly M.S. (2019). Effects of Short-Term In-Season Weightlifting Training on the Muscle Strength, Peak Power, Sprint Performance, and Ball-Throwing Velocity of Male Handball Players. J. Strength Cond. Res..

[55] Figueira B., Gonçalves B., Abade E., Paulauskas R., Masiulis N., Sampaio J. (2019). Effects of a 4-week combined sloped training program in young basketball players’ physical performance. Sci. Sport..

[56] Karim L. (2018). Weight Training for Speed and its Impact on the Skillful Performance of Basketball Player. J. Phys. Fitness Med. Treat. Sport..

[57] Afyon Y.A., Boyaci A. (2013). Investigation of the effects by compositely edited core-plyometric exercises in sedentary man on some physical and motoric parameters. Int. J. Acad. Res..

[58] Kumar A.D., Subramaniam P.K., Kumar P.P.S. (2014). Effect of Complex Training with Core Exercise Programme on Agility Among Male Soccer Players. Glob. J. Res. Anal..

[59] Mackenzie B. (2005). 101 Evaluation Tests.

[60] Majid N.C., Fauzi F. (2021). The Effect of Sprint Training on Vertical Jump Height of Female Youth Volleyball Players. Int. J. Hum. Mov. Sport. Sci..

[61] Wang Y., Lei S.-M., Wu C.-C. (2023). The Effect of Mindfulness Intervention on the Psychological Skills and Shooting Performances in Male Collegiate Basketball Athletes in Macau: A Quasi-Experimental Study. Int. J. Environ. Res. Public Health.

[62] Olteanu M., Oancea B.M., Badau D. (2023). Improving Effectiveness of Basketball Free Throws through the Implementation of Technologies in the Technical Training Process. Appl. Sci..

